# Dupilumab shows no elevated risk for maternal adverse pregnancy outcomes: A propensity‐matched cohort study

**DOI:** 10.1111/jdv.20670

**Published:** 2025-04-02

**Authors:** Sophie L. Preuß, Katja Bieber, Artem Vorobyev, Andreas Recke, Eva Lotta Moderegger, Henner Zirpel, Evelyn Gaffal, Diamant Thaçi, Khalaf Kridin, Ralf J. Ludwig

**Affiliations:** ^1^ Department of Dermatology University Medical Centre of the State of Schleswig‐Holstein (UKSH) Lübeck Germany; ^2^ Lübeck Institute of Experimental Dermatology University of Lübeck Lübeck Germany; ^3^ Institute and Comprehensive Centre for Inflammation Medicine University of Lübeck Lübeck Germany; ^4^ Azrieli Faculty of Medicine Bar‐Ilan University Safed Israel; ^5^ Unit of Dermatology and Skin Research Laboratory Barch Padeh Medical Center Poriya Israel

## Abstract

**Background:**

Type 2 chronic inflammatory diseases (T2IDs) are highly prevalent among women of reproductive age. Dupilumab, a monoclonal antibody, is increasingly used to treat T2IDs. While dupilumab is not approved during pregnancy, smaller studies suggest no increased risk of pregnancy complications (adverse pregnancy outcomes (APOs)). Additional data are required to better assess the drug's safety during pregnancy.

**Objectives:**

To retrospectively assess the risk of APOs in dupilumab‐treated pregnant women in a large real‐world database.

**Methods:**

Pregnant women with T2ID and dupilumab treatment during pregnancy were retrieved from the US Collaborative Network of TriNetX. Pregnant women with T2ID and without dupilumab treatment served as controls. Propensity score matching (PSM) for demographics, diagnoses, medications and putative APO risk factors was employed. Outcomes analysed included various maternal pregnancy complications, including premature obstetric labour, pregnancy‐induced hypertension, gestational diabetes, puerperal infections and spontaneous abortion. Survival analyses were assessed using the Kaplan–Meier method, outcome differences the log‐rank test and hazard ratios (HR) the Cox regression model.

**Results:**

During pregnancy, 293 women were exposed to dupilumab. Following PSM, no increased risks for APOs were noted. Of note, reduced risks for premature obstetric labour (HR: 0.11, confidence interval (CI): 0.03–0.45, *p* = 0.0002*)* and ‘any APO’ (HR: 0.53, CI: 0.33–0.84, *p* = 0.0067) in the dupilumab‐treated group were found. Furthermore, no difference in risks for any APO was noted between dupilumab‐treated and untreated women up to 6 months before pregnancy or during the postpartum period.

**Conclusions:**

This large‐scale propensity‐matched retrospective cohort study suggests a favourable safety profile of dupilumab during pregnancy. Given the difficulties of prospective studies during pregnancy, it provides valuable insights, though further studies are needed to confirm these findings and explore causal relationships.

## Graphical abstract

This large‐scale, propensity‐matched retrospective cohort study provides evidence that dupilumab treatment during pregnancy is not associated with an elevated risk of maternal adverse pregnancy outcomes (APOs). By utilizing real‐world data, it contributes critical insights into dupilumab's safety profile in pregnant women, addressing a crucial gap in clinical knowledge.

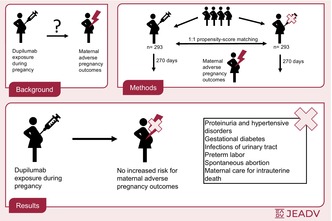

**Why was the study undertaken?**

- Pregnant women are often excluded from prospective clinical trials, leading to a significant lack of data on the safety of various treatments during pregnancy. This gap in knowledge complicates clinical decision‐making, particularly for conditions requiring long‐term therapy. To address this, the study aimed to assess the risk of adverse pregnancy outcomes (APOs) in pregnant women treated with dupilumab by analysing a large real‐world database.

**What does this study add?**

- This large‐scale, propensity‐matched retrospective cohort study provides evidence that dupilumab treatment during pregnancy is not associated with an increased risk of maternal adverse pregnancy outcomes (APOs). By leveraging real‐world data, the study offers valuable insights into the safety of dupilumab in pregnant women, filling a critical knowledge gap.

**What are the implications of this study for disease understanding and/or clinical care?**

- The study's findings provide an important contribution to the understanding of dupilumab's safety profile in pregnant women, suggesting no increased risk of adverse pregnancy outcomes. This evidence can help guide clinicians in making informed treatment decisions for pregnant patients requiring dupilumab, offering reassurance regarding its use in this setting. By addressing a crucial gap in clinical knowledge, the study supports safer and more confident prescribing practices, ultimately improving patient care for pregnant women with type 2 inflammatory diseases.

## INTRODUCTION

Around 20% of the general population is affected by chronic type 2 inflammatory diseases (T2IDs),^1^ many of which impact women of childbearing age.^2^, ^3^ During pregnancy, the immune system shifts towards Th2 dominance, often worsening or triggering T2IDs such as atopic eruption of pregnancy and bronchial asthma.^4^, ^5^ Effective treatment is crucial to reduce risks to both mother and child,^6^ yet systemic treatment options remain limited due to the lack of approved medications for pregnancy. As a result, many treatment decisions must rely on non‐licensed drugs and are off‐label, potentially leading to uncertainties for patients and medical practitioners.

While biologics like dupilumab are well‐documented in the general population, there is insufficient data on their safety during pregnancy, precluding clear recommendations and formal approval during this period.^7^, ^8^ Dupilumab, targeting IL‐4 and IL‐13 pathways, is approved for treating various T2IDs, including moderate to severe atopic dermatitis, severe bronchial asthma with type 2 reaction, severe chronic rhinosinusitis with nasal polyps, prurigo nodularis, eosinophilic esophagitis and recently as an add‐on maintenance treatment for adults with uncontrolled chronic obstructive pulmonary disease (COPD) characterized by raised blood eosinophils.

Even though there is beneficial data regarding the safety profile during pregnancy from smaller studies, case reports and series, the risk of dupilumab administration during pregnancy regarding adverse pregnancy outcomes (APOs) remains insufficiently explored. APOs refer to complications occurring during pregnancy, childbirth or after delivery, including preterm labour, hypertensive disorders, gestational diabetes mellitus, spontaneous abortion, intrauterine death and puerperal infections.

The largest study to date, by Avallone et al., involved 29 women exposed to dupilumab during pregnancy and reported no significant drug‐associated risk for adverse pregnancy or postpartum outcomes.^9^ Similarly, Escola et al. found no adverse maternal or fetal outcomes in 11 women treated during pregnancy and 2 during breastfeeding.^10^ Xara et al. also reported no APOs in six women treated with dupilumab.^11^ A large pharmacovigilance study and EMA findings indicated no APOs or effects on fertility or embryonic development.^12^, ^13^ Several case reports and a systematic review of 69 pregnancies also concluded that dupilumab exposure is associated with minimal risk for APOs.^7^, ^14^, ^15^, ^16^, ^17^, ^18^, ^19^, ^20^, ^21^, ^22^

APOs are significant public health concerns and require thorough investigation, especially when new therapies are introduced. With the increasing use of dupilumab for various T2IDs, understanding its effects during pregnancy is crucial, yet comprehensive data from larger cohort studies are lacking. To address this gap, we conducted a retrospective cohort study analysing the risk of maternal APOs in pregnant women with T2IDs treated with dupilumab using the TriNetX federated database.

## MATERIALS AND METHODS

### Study design and database

**FIGURE 1:**
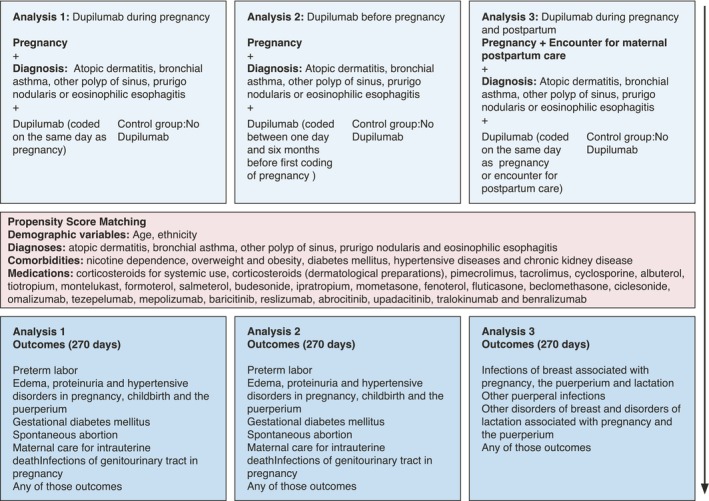
Graphical representation of the study design structure.

We conducted a propensity‐matched retrospective cohort study utilizing the US Collaborative Network of TriNetX, adhering to established protocols.^23^, ^24^, ^25^, ^26^ TriNetX is a company operating a federated healthcare database that provides real‐time access to electronic health records (EHRs) from healthcare organizations (HCOs). For this purpose, TriNetX partners with HCOs. Patient data are de‐identified to comply with HIPAA (in the United States) and GDPR (in the EU) regulations. Depending on the institution's policies and applied laws, patients may opt out of having their de‐identified data used for research. With over 110 million EHRs in the US Collaborative network, we identified cohorts of pregnant women diagnosed with diseases for which dupilumab is approved, both with and without treatment. The risk of APOs was then compared between these groups, analysing data for 270 days following the initial pregnancy diagnosis (Figure 1). Data for this study were collected in July 2024. This study was a collaboration between the University Clinic of Schleswig‐Holstein (UKSH) and TriNetX, allowing UKSH researchers to access the network.

### Ethics statement

Data accessible via TriNetX is presented in an aggregate form and only contains anonymized data as per the de‐identification standard defined by the US Health Insurance Portability and Accountability Act (HIPAA) in section §164,514(a). As this study exclusively used de‐identified anonymized electronic medical records, it did not require Institutional Review Board approval or written informed consent.

### Study population, definition of eligible patients, covariates for propensity matching and outcomes

EHRs from females aged 12 to 55 years in the US Collaborative Network of TriNetX were included (Figure 1). Pregnant women were identified by the following International Classification of Diseases, Tenth Revision, Clinical Modification (ICD10CM) or Procedure Coding System (ICD10PCS) codes and Current Procedural Terminology (CPT) codes: ICD10CM:Z32.01 (encounter for pregnancy test, result positive), ICD10CM:Z32.2 (encounter for childbirth instruction), ICD10CM:Z33 (pregnant state), ICD10CM:Z34 (encounter for supervision of a normal pregnancy), ICD10CM:Z36 (encounter for antenatal screening of mother), ICD10CM:Z3A (weeks of gestation), ICD10CM:Z03.7 (encounter for suspected maternal and fetal conditions ruled out), CPT:1010785 (diagnostic ultrasound procedures of the pelvis obstetrical), ICD10PCS:BY (fetus and obestrical) or ICD10PCS:10 (pregnancy). For postpartum analysis, the code ICD10CM:Z39 (encounter for maternal postpartum care and instruction) was also included.

To ensure a homogeneous study population, only patients receiving dupilumab for an approved indication were included. Diagnoses were selected to align as closely as possible with these indications. Therefore, pregnant women had to have at least one of the following diagnoses coded during pregnancy: Atopic dermatitis (ICD10CM:L20), bronchial asthma (ICD10CM:J45), other polyp of sinus (ICD10CM:J33.8), prurigo nodularis (ICD10CM:L28.1) and eosinophilic esophagitis (ICD10CM:K20.0). As dupilumab was approved for COPD only shortly before this study was conducted, patients with this diagnosis were not included in the analysis. Among these pregnant women with the indicated diagnoses, those treated with dupilumab were identified by RxNorm1876376 (dupilumab) (Figure 1).

The risk for maternal APOs was assessed in three settings: First, women were included if dupilumab treatment was coded on the same day as a pregnancy‐indicating diagnosis, with controls being women without dupilumab treatment from 6 months before to 9 months after this diagnosis. Second, women treated with dupilumab between 1 day and 6 months before the pregnancy‐indicating diagnosis were analysed, with controls being those without dupilumab treatment during the same timeframe. Third, for postpartum outcomes, women were included if dupilumab was coded on the same day as a pregnancy diagnosis or postpartum care, with controls following similar criteria (Figure 1).

EHRs with indicated dupilumab use were matched 1:1 with controls without dupilumab using PSM for demographic variables (age, ethnicity), diagnoses for which dupilumab is approved and comorbidities that may affect the risk for APOs, including nicotine dependence, overweight and obesity, diabetes mellitus, hypertensive diseases and chronic kidney disease.^27^, ^28^, ^29^, ^30^, ^31^, ^32^ Additionally, patients were matched for common medications approved for the indicated diagnoses, including corticosteroids for systemic use, corticosteroids (dermatological preparations), pimecrolimus, tacrolimus, cyclosporine, albuterol, tiotropium, montelukast, formoterol, salmeterol, budesonide, ipratropium, mometasone, fenoterol, fluticasone, beclomethasone, ciclesonide, omalizumab, tezepelumab, mepolizumab, baricitinib, reslizumab, abrocitinib, upadacitinib, tralokinumab and benralizumab.

Patients were analysed for the onset of the following outcomes within 270 days after coding of a pregnancy‐indicating diagnosis: Preterm labour (labour occurring between 20 and 37 weeks of gestation, ICD10CM:O60); oedema, proteinuria and hypertensive disorders in pregnancy, childbirth and the puerperium (ICD10CM:O11‐O16); gestational diabetes mellitus (ICD10CM:O24.4); spontaneous abortion (ICD10CM:O03); maternal care for intrauterine death (ICD10CM:O36.4); infections of the genitourinary tract in pregnancy (ICD10CM:O23) as well as any of the outcomes mentioned above (‘any APO’). For postpartum analysis, patients were analysed for infections of the breast associated with pregnancy, the puerperium and lactation (ICD10CM:O91); other puerperal infections (ICD10CM:O86) and other disorders of the breast and disorders of lactation associated with pregnancy and the puerperium (ICD10CM:O92) within 270 days after coding of a pregnancy‐indicating diagnosis or coding of an encounter for maternal postpartum care (Figure 1). For all analyses, patients with outcomes occurring prior to the respective index events were excluded. All outcomes were defined by ICD10CM codes prior to data collection.

In addition, the risk of APOs between pregnant women with T2ID treated with dupilumab and without T2ID (and without dupilumab treatment) was compared. Patients were matched 1:1 based on age, ethnicity and risk factors for APO, and assessed for APOs within 270 days of a pregnancy‐indicating diagnosis.

In a next analysis, the APO risk (independent of dupilumab) between pregnant women with and without T2IDs, further stratified by specific diseases, was analysed. Pregnant women with at least one T2ID or specific T2ID were matched in the same way 1:1 to controls without T2ID, and APO occurrence was assessed as described above.

### Statistical analysis

Statistical analyses were performed using the TriNetX platform. Baseline characteristics were described using means and standard deviations or frequencies and percentages. Variables were compared with Student's *t*‐test for continuous and Pearson chi‐square test for dichotomous variables. Survival analyses were conducted using Kaplan–Meier and the log‐rank test. Hazard ratios (HR) were estimated with Cox regression. The Bonferroni method adjusted α = 0.05 for seven APO categories, using 0.0071 per category.

## RESULTS

### Study population

For the primary analysis, 293 EHRs with dupilumab exposure during pregnancy and T2ID were retrieved, matched with an equally sized non‐dupilumab group. The mean age was 31.70 ± 9.26 years for the dupilumab group and 31.97 ± 9.09 years for the control group. For the analysis of dupilumab use up to 6 months before pregnancy, 243 EHRs were retrieved for each group, with mean ages of 29.9 ± 7.63 and 29.8 ± 8.41 years, respectively. For postpartum outcomes, 300 EHRs per group were retrieved, with mean ages of 31.6 ± 9.10 and 31.7 ± 9.32 years. In all analyses, no significant differences were found in demographics, comorbidities or medications after PSM (Table 1, Tables S1–S7).

**TABLE 1:** Analysis of dupilumab treatment during pregnancy. Baseline characteristics of the cohorts before propensity‐score matching and below after propensity‐score matching. Percentage values refer to the respective groups.

Before matching, *After matching*	Dupilumab	Controls	Std diff.
Demographics (*n*, %)			
*n*	299 *293*	532,774 *293*	‐
Age in years (mean, SD)	31.60 ± 9.3 *31.70 ± 9.26*	27.50 ± 7.23 *31.97 ± 9.09*	0.5265 *0.0182*
White	197 (65.89%) *193 (65.87%)*	304,608 (57.17%) *194 (66.21%)*	0.1787 *0.0072*
Not Hispanic or Latino	247 (82.61%) *241 (82.25%)*	361,397 (67.83%) *246 (83.96%)*	0.3474 *0.0456*
Black or African American	63 (21.07%) *62 (21.16%)*	114,493 (21.49%) *53 (18.09%)*	0.0103 *0.0774*
Diagnoses (*n*, %)			
Atopic dermatitis (L20)	96 (32.11%) *92 (31.40%)*	5435 (1.02%) *98 (33.45%)*	0.9205 *0.0438*
Bronchial asthma (J45)	151 (50.50%) *145 (49.49%)*	93,747 (17.60%) *150 (51.20%)*	0.7405 *0.0341*
Other polyp of sinus (J33.8)	10 (3.34%) *10 (3.41%)*	184 (0.04%) *10 (3.41%)*	0.2590 *<0.0001*
Prurigo nodularis (L28.1)	10 (3.34%) *10 (3.41%)*	427 (0.08%) *10 (3.41%)*	0.2536 *<0.0001*
Eosinophilic esophagitis (K20.0)	25 (8.36%) *23 (7.85%)*	924 (0.17%) *25 (8.53%)*	0.4137 *0.0249*
Comorbidity (*n*, %)			
Overweight and obesity (E66)	74 (24.75%) *69 (23.55%)*	32,921(6.19%) *68 (23.21%)*	0.5311 *0.0081*
Nicotine dependence (F17)	50 (16.72%) *46 (15.70%)*	27,905 (5.24%) *44 (15.02%)*	0.3737 *0.0189*
Hypertensive diseases (I10‐I1A)	63 (21.07%) *60 (20.48%)*	20,678 (3.89%) *57 (19.45%)*	0.5387 *0.0256*
Diabetes mellitus (E08‐E13)	57 (19.06%) *53 (18.09%)*	14,931 (2.80%) *51 (17.41%)*	0.5397 *0.0179*
Chronic kidney disease (N18)	18 (6.02%) 17 (5.80%)	1993 (0.37%) 17 (5.80%)	0.3252 <0.0001
Medications (*n*, %)			
Corticosteroids for systemic use	218 (72.91%) *213 (72.70%)*	78,189 (14.68%) *227 (77.47%)*	1.4497 *0.1106*
Corticosteroids, dermatological preparations	200 (66.89%) 194 (66.21%)	66,156 (12.42%) 207 (70.65%)	1.3406 0.0956
Pimecrolimus	16 (5.35%) *14 (4.79%)*	554 (0.10%) *22 (7.51%)*	0.3264 *0.1139*
Tacrolimus	34 (11.37%) *33 (11.26%)*	1085 (0.20%) *38 (12.97%)*	0.4925 *0.0523*
Cyclosporine	12 (4.01%) *11 (3.75%)*	374 (0.07%) *10 (3.41%)*	0.2816 *0.0184*
Albuterol	115 (38.46%) *110 (37.54%)*	65,698 (12.33%) *111 (37.89%)*	0.6293 *0.0070*
Tiotropium	27 (9.03%) *24 (8.19%)*	671 (0.13%) *16 (5.46%)*	0.4360 *0.1084*
Montelukast	78 (26.09%) *74 (25.26%)*	712,929 (2.43%) *67 (22.87%)*	0.7191 *0.0559*
Formoterol	45 (15.05%) *43 (14.68%)*	5409 (1.02%) *42 (14.33%)*	0.5354 *0.0097*
Salmeterol	36 (12.04%) *33 (11.26%)*	7407 (1.39%) *25 (8.53%)*	0.4355 *0.0915*
Budesonide	71 (23.75%) *67 (22.87%)*	7863 (1.48%) *57 (19.45%)*	0.7121 *0.0836*
Ipratropium	37 (12.38%) *35 (11.95%)*	10,523 (1.98%) *40 (13.65%)*	0.4114 *0.0511*
Mometasone	40 (13.38%) *39 (13.31%)*	4606 (0.87%) *43 (14.68%)*	0.5016 *0.0394*
Fenoterol	0 *0*	0 *0*	‐ ‐
Fluticasone	96 (32.11%) *92 (31.40%)*	31,384 (5.89%) *81 (27.65%)*	0.6922 *0.0824*
Beclomethasone	10 (3.34%) *10 (3.41%)*	2666 (0.5%) *10 (3.41%)*	0.2082 *<0.0001*
Ciclesonide	0 *0*	90 (0.02%) *0*	0.0184 ‐
Omalizumab	10 (3.34%) *10 (3.41%)*	266 (0.05%) *10 (3.41%)*	0.2572 *<0.0001*
Tezepelumab	10 (3.34%) *10 (3.41%)*	10 (0.002%) *0*	0.2628 *0.2658*
Mepolizumab	10 (3.34%) *10 (3.41%)*	54 (0.01%) *10 (3.41%)*	0.2619 *<0.0001*
Baricitinib	10 (3.34%) *10 (3.41%)*	10 (0.002%) *0*	0.2628 *0.2658*
Reslizumab	0 *0*	0 *0*	‐ ‐
Abrocitinib	0 *0*	10 (0.002%) *0*	0.0061 ‐
Upadacitinib	10 (3.34%) *0*	18 (0.003%) *10 (3.41%)*	0.2627 *0.2658*
Tralokinumab	10 (3.34%) *0*	10 (0.002%) *0*	0.2628 ‐
Benralizumab	10 (3.34%) *10 (3.41%)*	31 (0.006%) *10 (3.41%)*	0.2624 *<0.0001*

### No increased risk for any analysed APO in patients treated with dupilumab during pregnancy

**FIGURE 2:**
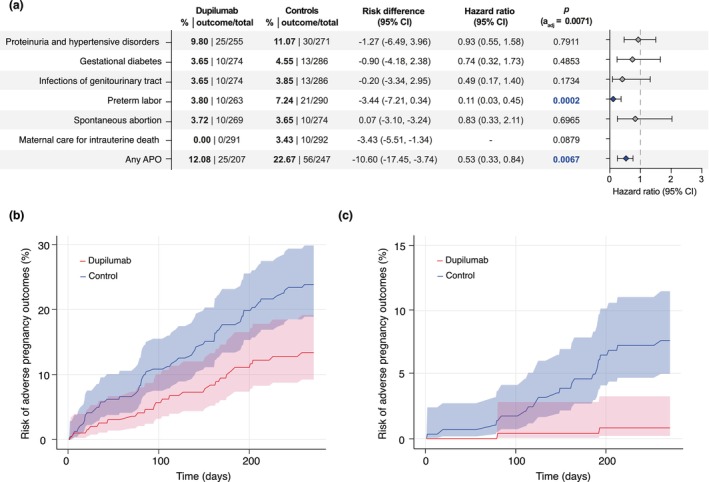
(a) Risk of adverse pregnancy outcomes (APOs) in patients with dupilumab treatment during pregnancy and controls. Points indicate hazard ratios (HR), error bars correspond to the confidence interval (CI). Patients with the outcome prior to the index event were excluded, resulting in different group sizes for each outcome. *To protect patient privacy, numbers are rounded up to 10. (b) Nelson Aalen Plot contrasting the risk of ‘any APO’ in patients with dupilumab treatment during pregnancy and controls. Outcomes up to 270 days after the index event (pregnancy with or without dupilumab treatment) are shown. Data are based on 293 electronic health records (EHRs) for cases and propensity‐matched controls. (c) Nelson Aalen Plot contrasting the risk of preterm labour in patients with dupilumab treatment during pregnancy and controls. Outcomes up to 270 days after the index event (pregnancy with or without dupilumab treatment) are shown. Data are based on 293 EHRs for cases and propensity‐matched controls.

We document that, compared to pregnancies without dupilumab, there was no higher risk for any analysed specific APO or the combined group (any APO) in the group where dupilumab treatment was coded on the same day as a pregnancy‐indicating diagnosis. Of note, there was a significantly reduced risk for preterm labour in the dupilumab‐exposed group, where the risk amounted to 3.80%, as opposed to 7.24% in the non‐dupilumab‐exposed group (HR:0.11, (confidence interval) CI:0.03–0.45, *p* = 0.0002). Additionally, the risk for any of the analysed APOs was significantly lower in the dupilumab‐treated group at 12.08% compared to 22.67% in the control group (HR:0.53, CI:0.33–0.84, *p* = 0.0067). No significant differences between the two groups were observed for the risk of proteinuria and hypertensive disorders (HR:0.93, CI:0.55–1.58, *p* = 0.7911), gestational diabetes (HR:0.74, CI:0.32–1.73, *p* = 0.4853), infections of the genitourinary tract (HR:0.49, CI:0.17–1.40, *p* = 0.1734), spontaneous abortion (HR:0.83, CI:0.33–2.11, *p* = 0.6965) and maternal care for intrauterine death (HR:‐, CI:‐, *p* = 0.879, Figure 2).

### No increased risk for APOs in patients with dupilumab treatment during pregnancy compared to patients without T2ID

**FIGURE 3:**
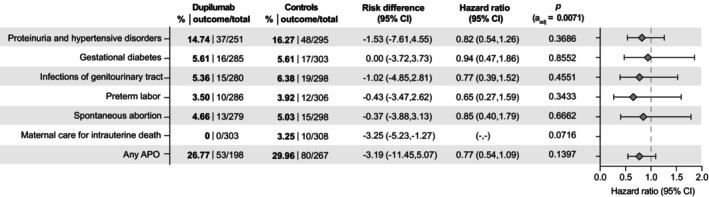
Risk of adverse pregnancy outcomes (APOs) in patients with dupilumab treatment and T2ID compared to controls without dupilumab treatment and without T2ID. Points indicate hazard ratios (HR), error bars correspond to the confidence interval (CI). Patients with the outcome prior to the index event were excluded. Data are based on 283 electronic health records (EHRs) for cases and propensity‐matched controls. *To protect patient privacy, numbers are rounded up to 10.

To better contextualize the observed risk reduction in pregnant women with T2IDs exposed to dupilumab during pregnancy, additional comparisons were conducted: (1) Assessing the risk in pregnant women with T2IDs exposed to dupilumab versus those without T2IDs and without dupilumab exposure, and (2) comparing pregnant women with T2IDs to those without T2IDs. In the analysis of the risk of APOs between pregnant women with T2ID who were treated with dupilumab and pregnant women without T2ID (and without dupilumab exposure) no significant differences were observed for specific APOs or ‘any APO’ (HR:0.77, CI:0.54–1.09, *p* = 0,1397, Figure 3).

### Increased risk for APOs in patients with T2ID compared to patients without T2ID

In the second analysis, the risk of APOs (independent of dupilumab treatment) was compared between women with and without T2IDs. In addition, comparisons between pregnant women diagnosed with one T2ID and non‐T2ID controls relating to their APO risk were performed.

**FIGURE 4:**
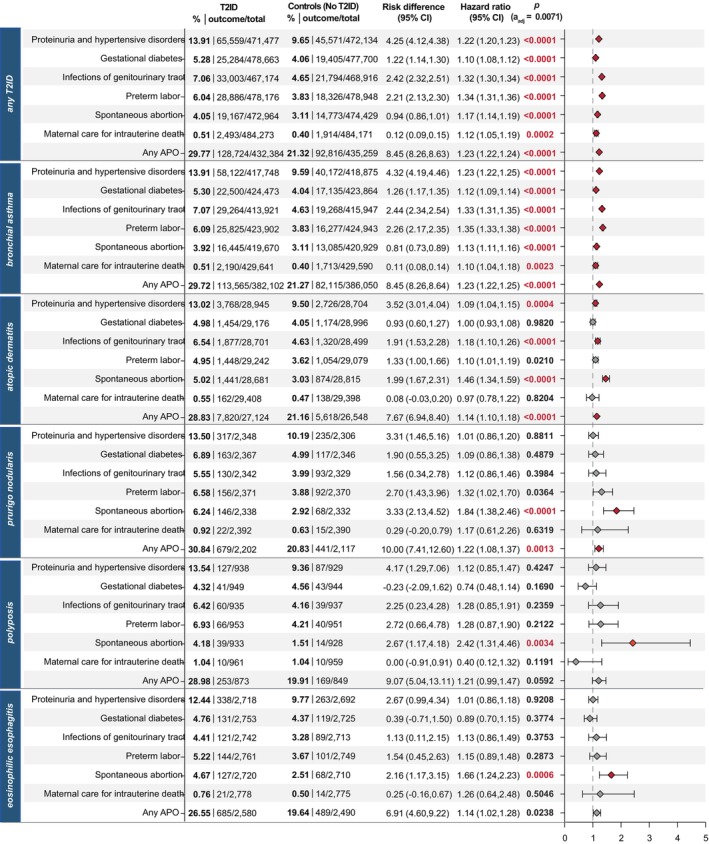
Risk of adverse pregnancy outcomes (APOs) in patients with T2IDs compared to controls without any T2ID. Points indicate hazard ratios (HR), error bars correspond to the confidence interval (CI). Patients with the outcome prior to the index event were excluded.

Patients with one or more T2IDs had a significantly increased risk for each analysed APO as well as for ‘any APO’ (HR:1.23, CI:1.22–1.24, *p* < 0.0001). A similar pattern was observed in patients with bronchial asthma, who also exhibited significantly increased risks for each analysed APO and ‘any APO’ (HR:1.23, CI:1.22–1.25, *p* < 0.0001). Patients with atopic dermatitis had a significantly increased risk for proteinuria and hypertensive disorders, spontaneous abortion, infections of the genitourinary tract and ‘any APO’ (HR:1.14, CI:1.10–1.18, *p* < 0.0001). Similarly, patients with prurigo nodularis had a significantly increased risk of spontaneous abortion and ‘any APO’ (HR:1.22, CI:1.08–1.37, *p* = 0.0013) compared to controls. Patients with polyposis nasi and eosinophilic esophagitis had a higher risk for spontaneous abortion compared to the non‐T2ID control group (Figure 4).

### No increased risk for any analysed APOs in patients with dupilumab treatment up to 6 months before pregnancy

**FIGURE 5:**
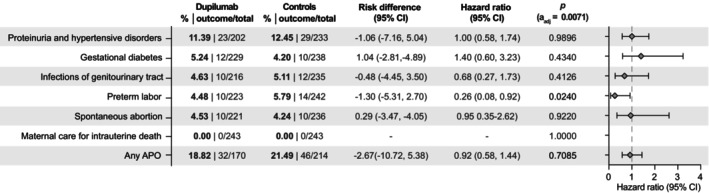
Risk of adverse pregnancy outcomes (APOs) in patients with dupilumab treatment 6 months to 1 day before pregnancy and controls. Points indicate hazard ratios (HR), error bars correspond to the confidence interval (CI). Patients with the outcome prior to the index event were excluded. Data are based on 243 electronic health records (EHRs) for cases and propensity‐matched controls. *To protect patient privacy, numbers are rounded up to 10.

There were no significant differences in the risk for specific or any analysed APO (HR:0.92, CI:0.58–1.44, *p* = 0.7085) in the group where dupilumab treatment was coded up to 6 months before a pregnancy‐indicating diagnosis compared to non‐treated controls (Figure 5).

### No increased risk for postpartum APOs in patients with dupilumab treatment during pregnancy or postpartum period

**FIGURE 6:**
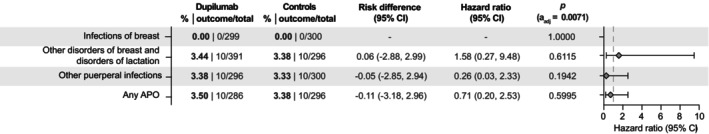
Risk of adverse pregnancy outcomes (APOs) in patients with dupilumab treatment during pregnancy or postpartum and controls. Points indicate hazard ratios (HR), error bars correspond to the confidence interval (CI). Patients with the outcome prior to the index event were excluded. Data are based on 300 electronic health records (EHRs) for cases and propensity‐matched controls. *To protect patient privacy, numbers are rounded up to 10.

In both groups, there were no records found with infections of the breast associated with pregnancy, the puerperium and lactation (HR:‐, CI:‐, *p* = 1.000). Compared to the group without dupilumab treatment, there were no significant differences in the risk of the group that was treated during pregnancy or the postpartum period with dupilumab regarding other disorders of the breast and disorders of lactation (HR 1.58, CI 0.27–9.48, *p* = 0.6115), other puerperal infections (HR:0.26, CI:0.03–2.33, *p* = 0.1942) and any analysed APO (HR:0.71, CI:0.20–2.53, *p* = 0.5995, Figure 6).

## DISCUSSION

Pregnant and lactating women are often excluded from interventional clinical trials due to safety concerns for both mother and fetus.^33^, ^34^ As a result, clinicians rely on observational studies to assess medication safety during these periods. To ensure comprehensive safety assessments, it is essential to analyse large patient populations. While no significant risk for APOs associated with off‐label dupilumab use during pregnancy has been reported, uncertainty persists due to limited case data. The strength of this study lies in its large sample size, including 243 women treated before pregnancy, 293 treated during pregnancy and 300 treated during pregnancy and postpartum with T2IDs.

We found in no analysis a significantly increased risk for any analysed APO in dupilumab‐treated groups. These results align with data from previous small‐scale studies and case reports, indicating a favourable safety profile during pregnancy.^9^, ^11^, ^13^, ^14^, ^15^, ^16^, ^17^, ^19^, ^20^, ^35^ Regarding the risk for infections of the genitourinary tract, results are consistent with a recent study showing that dupilumab treatment for moderate‐to‐severe atopic dermatitis was not associated with an increased risk of systemic or cutaneous infections.^36^

We observed a significantly reduced risk for “any APO” and preterm labour in dupilumab‐treated patients compared to controls. In contrast, Avallone et al. reported an increased incidence of preterm births among 29 dupilumab‐exposed patients, suggesting a possible rebound in immune system activation after discontinuation of the drug.^9^ Information on dupilumab continuation or discontinuation during pregnancy was not available in our study. The apparent discrepancy may be due to patients in our study who continued dupilumab throughout pregnancy. Despite the limitations of our retrospective cohort design, findings from 293 patients provide a more robust assessment. Further investigation is needed to explore potential causal effects of dupilumab on preterm labour.

In general, it is important to consider whether the reduced risks for ‘any APO’ and preterm labour with dupilumab treatment reflect a normalization of APO risk, potentially mitigating an elevated baseline risk linked to T2ID. The presence of such a baseline risk remains controversial and varies by disease. In more detail, bronchial asthma has been associated with an increased risk of hypertensive pregnancy disorders, preterm birth and abortion,^37^, ^38^, ^39^ aligning with our findings of significantly higher risks for all specific APOs and ‘any APO’ in asthma patients. Studies on atopic dermatitis and APOs show conflicting results. While some reported a lower risk of preterm birth and stillbirth,^40^, ^41^ others found increased risks of threatened abortion, preeclampsia/eclampsia and preterm labour.^42^ In our study, atopic dermatitis was associated with a significantly higher risk of proteinuria and hypertensive disorders, spontaneous abortion, genitourinary infections, and ‘any APO’, but not preterm labour. For other T2IDs, data on APO risk are scarce. We observed that prurigo nodularis, polyposis nasi and eosinophilic esophagitis were linked to a higher risk of spontaneous abortion. In patients with at least one T2ID, an overall increased risk for all specific APOs and ‘any APO’ was observed, suggesting an elevated baseline risk. However, stratified analyses showed this risk varied by disease. To assess whether dupilumab treatment normalizes this risk, we compared dupilumab‐treated patients with T2IDs to pregnant women without T2IDs. No significant differences were found for any APOs, suggesting dupilumab may mitigate the increased baseline risk. However, this does not imply causality, and further research is needed to distinguish the effects of the underlying disease from those of dupilumab treatment.

Interestingly, the significant risk reduction of preterm labour and ‘any APO’ with dupilumab treatment during pregnancy was no longer detected when treatment occurred from 6 months before to 1 day before a pregnancy‐indicating diagnosis. Whether the absence of a risk reduction is caused by a lack of positive effects of dupilumab treatment during pregnancy, such as improved disease control, requires further prospective studies.

In the analysis of postpartum outcomes, no significant differences were found between the dupilumab‐treated and non‐treated groups regarding the risk for infections of the breast, other puerperal infections or other disorders of the breast and disorders of lactation. These findings align with results from previous smaller studies indicating no complications in lactation.^11^, ^16^, ^19^

The TriNetX dataset allows detailed propensity score matching (PSM) to minimize bias from confounders. However, several limitations should be noted. First, the retrospective design may introduce biases and data collection limitations. Second, diagnostic codes may contain inaccuracies. Nonetheless, the inclusion of female sex, age, pregnancy‐related diagnoses and approved dupilumab indications reduces this risk. Third, while the study included women who received at least one dose of dupilumab, the rate of discontinuation or frequency of administration could not be determined. Fourth, only maternal diagnoses were analysed, excluding APOs in the fetus's record due to privacy protocols. Lastly, the severity of diagnoses could not be compared.

Despite limitations, our study's findings warrant clinical consideration until prospective data become available. This is the first large cohort study comparing the risk of APOs in pregnant women with and without dupilumab treatment. We found no increased risk and even a reduced risk for some APOs in the dupilumab‐treated group, supporting its safety for maternal APOs. However, as dupilumab is not approved for pregnancy, potential risks and benefits should be weighed in shared decision‐making with healthcare professionals.

## AUTHOR CONTRIBUTIONS

SLP and RJL were responsible for conceptualization, methodology, investigation and writing the original draft. KB, AV and AR contributed to data curation, formal analysis and visualization, writing, review and editing. ELM, HZ, EG, DT and KK contributed to writing, review and editing. All authors provided resources, participated in validation and read and approved the final version of the manuscript.

## FUNDING INFORMATION

Cluster of Excellence ‘Precision Medicine in Chronic Inflammation’ (EXC 2167) and Collaborative Research Centre ‘PANTAU’ (SFB 1526), Individual Research Grant LU 877/25–1, all from the Deutsche Forschungsgemeinschaft and the Schleswig‐Holstein Excellence‐Chair Program from the State of Schleswig Holstein. The funders had no role in the study design, data collection, data analyses, interpretation or writing of the report.

## CONFLICT OF INTEREST STATEMENT

The authors declare that this study was designed and conducted in the absence of any financial or commercial relationships that could be construed as a potential conflict of interest. SP received support for travel from TriNetX unrelated to the current work. HZ received support for meeting attendance and/or travel from Pfizer, UCB Pharma, Almirall, Janssen and TriNetX unrelated to the current work.

## ETHICAL APPROVAL

Data accessible via TriNetX is presented in an aggregate form and only contains anonymized data as per the de‐identification standard defined by the US Health Insurance Portability and Accountability Act (HIPAA) in section §164,514(a). As this study exclusively used de‐identified anonymized electronic medical records, it did not require Institutional Review Board approval.

## ETHICS STATEMENT

As this study exclusively used de‐identified anonymized electronic medical records, it did not require written informed consent.

## Supporting information


Data S1.

## Data Availability

The data that support the findings of this study are available on request from the corresponding author, SLP.
