# Supplementary material for: Dupilumab shows no elevated risk for maternal adverse pregnancy outcomes: A propensity‐matched cohort study
Source: J Eur Acad Dermatol Venereol. 2025 Apr 2;39(9):1576–87. doi: 10.1111/jdv.20670 (PMC12376243; doi:10.1111/jdv.20670)
Supplement: Supplementary file 1 — Data S1. [file JDV-39-1576-s001.docx]

**Supplemental Material**

|  | **Before matching** | | | | | **After matching** | | | | |
| --- | --- | --- | --- | --- | --- | --- | --- | --- | --- | --- |
|  | Dupilumab (n) | Dupilumab (%) | No T2ID (n) | No T2ID (%) | Std. Diff. | Dupilumab (n) | Dupilumab (%) | No T2ID (n) | No T2ID (%) | Std. Diff. |
| ***Demographics (n, %)*** | | | | | | | | | | |
| n | 283 |  | 4198386 |  |  | 283 |  | 283 |  |  |
| Age at Index (mean, SD) | 29.70 ± 6.35 |  | 28.50 ± 6.29 |  | 0.2018 | 29.70 ± 6.35 |  | 29.70 ± 6.33 |  | 0.0010 |
| Not Hispanic or Latino | 224 | 79.152% | 2420027 | 57.642% | 0.4755 | 224 | 79.152% | 224 | 79.152% | 0 |
| White | 143 | 50.53% | 2241200 | 53.382% | 0.0571 | 143 | 50.53% | 143 | 50.53% | 0 |
| Black or African American | 91 | 32.155% | 732049 | 17.436% | 0.3459 | 91 | 32.155% | 90 | 31.802% | 0.0076 |
| ***Comorbidities (n, %)*** |  |  |  |  |  |  |  |  |  |  |
| Overweight and obesity (E66) | 82 | 28.975% | 143714 | 3.423% | 0.7394 | 82 | 28.975% | 82 | 28.975% | 0 |
| Hypertensive diseases (I10-I1A) | 34 | 12.014% | 62864 | 1.497% | 0.4285 | 34 | 12.014% | 34 | 12.014% | 0 |
| Nicotine dependence (F17) | 42 | 14.841% | 94288 | 2.246% | 0.4625 | 42 | 14.841% | 42 | 14.841% | 0 |
| Diabetes mellitus (E08-E13) | 17 | 6.007% | 36528 | 0.87% | 0.2847 | 17 | 6.007% | 18 | 6.36% | 0.0147 |
| Chronic kidney disease (N18) | 10 | 3.534% | 4482 | 0.107% | 0.2585 | 10 | 3.534% | 10 | 3.534% | 0 |

**Supplemental Table 1:** Baseline characteristics of analysis of patients with T2ID (type two inflammatory disease) and dupilumab treatment and patients without T2ID. Baseline characteristics of the cohorts before propensity-score matching and after propensity-score matching. Percentage values refer to the respective groups.

|  | **Before matching** | | | | | **After matching** | | | | |
| --- | --- | --- | --- | --- | --- | --- | --- | --- | --- | --- |
|  | T2ID (n) | T2ID (%) | No T2ID (n) | No T2ID (%) | Std. Diff. | T2ID (n) | T2ID (%) | No T2ID (n) | No T2ID (%) | Std. Diff. |
| ***Demographics (n, %)*** | | | | | | | | | | |
| n | 516235 |  | 4311027 |  |  | 485033 |  | 485033 |  |  |
| Age at Index (mean, SD) | 27.3 ± 6.53 |  | 28.40 ± 6.33 |  | 0.2018 | 27.3 ± 6.53 |  | 27.3 ± 6.53 |  | 0.0010 |
| Not Hispanic or Latino | 338510 | 69.78% | 2289018 | 56.566 % | 0.2766 | 338436 | 69.776 % | 338527 | 69.795 % | 0.0004 |
| White | 265697 | 54.771 % | 2169123 | 53.603 % | 0.0234 | 265675 | 54.775 % | 265733 | 54.787 % | 0.0002 |
| Black or African American | 125135 | 25.795 % | 684965 | 16.927 % | 0.2177 | 125083 | 25.789 % | 125138 | 25.8% | 0.0003 |
| ***Comorbidities (n, %)*** |  |  |  |  |  |  |  |  |  |  |
| Overweight and obesity (E66) | 52550 | 10.833 % | 140537 | 3.473 % | 0.2885 | 52476 | 10.819 % | 52474 | 10.819 % | <0.0001 |
| Hypertensive diseases (I10-I1A) | 21746 | 4.483 % | 60786 | 1.502 % | 0.1756 | 21672 | 4.468 % | 21682 | 4.47% | 0.0001 |
| Nicotine dependence (F17) | 38437 | 7.923 % | 94108 | 2.326 % | 0.2559 | 38363 | 7.909 % | 38323 | 7.901 % | 0.0003 |
| Diabetes mellitus (E08-E13) | 11914 | 2.456 % | 36594 | 0.904 % | 0.1209 | 11850 | 2.443 % | 11720 | 2.416 % | 0.0017 |
| Chronic kidney disease (N18) | 1557 | 0.321 % | 4357 | 0.108 % | 0.0461 | 1550 | 0.32% | 1406 | 0.29% | 0.0054 |

**Supplemental Table 2:** Baseline characteristics of analysis of patients with **any T2ID** (type two inflammatory disease) and patients without T2ID. Baseline characteristics of the cohorts before propensity-score matching and after propensity-score matching. Percentage values refer to the respective groups.

|  | **Before matching** | | | | | **After matching** | | | | |
| --- | --- | --- | --- | --- | --- | --- | --- | --- | --- | --- |
|  | Asthma (n) | Asthma (%) | No T2ID (n) | No T2ID (%) | Std. Diff. | Asthma (n) | Asthma (%) | No T2ID (n) | No T2ID (%) | Std. Diff. |
| ***Demographics (n, %)*** | | | | | | | | | | |
| n | 456064 |  | 4311027 |  |  | 430350 |  | 430350 |  |  |
| Age at Index (mean, SD) | 27.4 ± 6.49 |  | 28.5 ± 6.29 |  | 0.2018 | 27.4 ± 6.49 |  | 27.3 ± 6.48 |  | 0.0010 |
| Not Hispanic or Latino | 297612 | 69.15% | 2289018 | 56.566% | 0.2627 | 297577 | 69.148% | 297674 | 69.17% | 0.0005 |
| White | 237856 | 55.266% | 2169123 | 53.603% | 0.0334 | 237849 | 55.269% | 237817 | 55.261% | 0.0001 |
| Black or African American | 110012 | 25.561% | 684965 | 16.927% | 0.2123 | 109983 | 25.557% | 110087 | 25.581% | 0.0006 |
| ***Comorbidities (n,%)*** | | | | | | | | | | |
| Overweight and obesity (E66) | 44371 | 10.31% | 140537 | 3.473% | 0.2724 | 44335 | 10.302% | 44384 | 10.313% | 0.0004 |
| Nicotine dependence (F17) | 34933 | 8.117% | 94108 | 2.326% | 0.2626 | 34897 | 8.109% | 34766 | 8.079% | 0.0011 |
| Hypertensive diseases (I10-I1A) | 19007 | 4.416% | 60786 | 1.502% | 0.1726 | 18971 | 4.408% | 18958 | 4.405% | 0.0001 |
| Diabetes mellitus (E08-E13) | 10530 | 2.447% | 36594 | 0.904% | 0.1204 | 10498 | 2.439% | 10379 | 2.412% | 0.0018 |
| Chronic kidney disease (N18) | 1324 | 0.308% | 4357 | 0.108% | 0.0439 | 1320 | 0.307% | 1205 | 0.28% | 0.0049 |

**Supplemental Table 3:** Baseline characteristics of analysis of patients with **bronchial asthma** and patients without T2ID. Baseline characteristics of the cohorts before propensity-score matching and after propensity-score matching. Percentage values refer to the respective groups.

|  | **Before matching** | | | | | **After matching** | | | | |
| --- | --- | --- | --- | --- | --- | --- | --- | --- | --- | --- |
|  | Atopic dermatitis (n) | Atopic dermatitis (%) | No T2ID (n) | No T2ID (%) | Std. Diff. | Atopic dermatitis (n) | Atopic dermatitis (%) | No T2ID (n) | No T2ID (%) | Std. Diff. |
| ***Demographics (n, %)*** | | | | | | | | | | |
| n | 32024 |  | 4311027 |  |  | 29438 |  | 29438 |  |  |
| Age at Index (mean, SD) | 27.2 ± 6.75 |  | 28.5 ± 6.29 |  | 0.2018 | 27.2 ± 6.75 |  | 28.5 ± 6.29 |  | 0.0010 |
| Not Hispanic or Latino | 21539 | 73.162% | 2289018 | 56.566% | 0.3530 | 21537 | 73.161% | 21543 | 73.181% | 0.0005 |
| White | 14028 | 47.649% | 2169123 | 53.603% | 0.1193 | 14028 | 47.653% | 14031 | 47.663% | 0.0002 |
| Black or African American | 8078 | 27.439% | 684965 | 16.927% | 0.2551 | 8076 | 27.434% | 8076 | 27.434% | 0 |
| ***Comorbidities (n,%)*** | | | | | | | | | | |
| Overweight and obesity (E66) | 3735 | 12.687% | 140537 | 3.473% | 0.3430 | 3733 | 12.681% | 3732 | 12.677% | 0.0001 |
| Nicotine dependence (F17) | 1456 | 4.946% | 94108 | 2.326% | 0.1403 | 1455 | 4.943% | 1456 | 4.946% | 0.0002 |
| Hypertensive diseases (I10-I1A) | 1187 | 4.032% | 60786 | 1.502% | 0.1547 | 1185 | 4.025% | 1183 | 4.019% | 0.0003 |
| Diabetes mellitus (E08-E13) | 508 | 1.726% | 36594 | 0.904% | 0.0722 | 507 | 1.722% | 500 | 1.698% | 0.0018 |
| Chronic kidney disease (N18) | 89 | 0.302% | 4357 | 0.108% | 0.0430 | 87 | 0.296% | 78 | 0.265% | 0.0058 |

**Supplemental Table 4:** Baseline characteristics of analysis of patients with **atopic dermatitis** and patients without T2ID. Baseline characteristics of the cohorts before propensity-score matching and after propensity-score matching. Percentage values refer to the respective groups.

|  | **Before matching** | | | | | **After matching** | | | | |
| --- | --- | --- | --- | --- | --- | --- | --- | --- | --- | --- |
|  | Prurigo nodularis (n) | Prurigo nodularis (%) | No T2ID (n) | No T2ID (%) | Std. Diff. | Prurigo nodularis (n) | Prurigo nodularis (%) | No T2ID (n) | No T2ID (%) | Std. Diff. |
| ***Demographics (n, %)*** | | | | | | | | | | |
| n | 2395 |  | 4311027 |  |  | 2395 |  | 2394 |  |  |
| Age at Index (mean, SD) | 30.3 ± 6.24 |  | 28.5 ± 6.29 |  | 0.2018 | 30.3 ± 6.23 |  | 30.3 ± 6.23 |  | 0.0010 |
| Not Hispanic or Latino | 1718 | 71.733% | 2289018 | 56.566% | 0.32028732 | 1717 | 71.721% | 1723 | 71.972% | 0.0056 |
| White | 1408 | 58.789% | 2169123 | 53.603% | 0.10466291 | 1408 | 58.814% | 1404 | 58.647% | 0.0034 |
| Black or African American | 470 | 19.624% | 684965 | 16.927% | 0.06983759 | 469 | 19.591% | 472 | 19.716% | 0.0032 |
| ***Comorbidities (n,%)*** | | | | | | | | | | |
| Overweight and obesity (E66) | 264 | 11.023% | 140537 | 3.473% | 0.29432726 | 263 | 10.986% | 257 | 10.735% | 0.0081 |
| Nicotine dependence (F17) | 136 | 5.678% | 94108 | 2.326% | 0.17168899 | 136 | 5.681% | 136 | 5.681% | 0 |
| Hypertensive diseases (I10-I1A) | 151 | 6.305% | 60786 | 1.502% | 0.24989988 | 150 | 6.266% | 148 | 6.182% | 0.0035 |
| Diabetes mellitus (E08-E13) | 119 | 4.969% | 36594 | 0.904% | 0.24250458 | 118 | 4.929% | 120 | 5.013% | 0.00384 |
| Chronic kidney disease (N18) | 18 | 0.752% | 4357 | 0.108% | 0.09856797 | 17 | 0.71% | 18 | 0.752% | 0.0049 |

**Supplemental Table 5:** Baseline characteristics of analysis of patients with **prurigo nodularis** and patients without T2ID. Baseline characteristics of the cohorts before propensity-score matching and after propensity-score matching. Percentage values refer to the respective groups.

|  | **Before matching** | | | | | **After matching** | | | | |
| --- | --- | --- | --- | --- | --- | --- | --- | --- | --- | --- |
|  | Other polyp of sinus (n) | Other polyp of sinus (%) | No T2ID (n) | No T2ID (%) | Std. Diff. | Other polyp of sinus (n) | Other polyp of sinus (%) | No T2ID (n) | No T2ID (%) | Std. Diff. |
| ***Demographics (n, %)*** | | | | | | | | | | |
| n | 961 |  | 4311027 |  |  | 961 |  | 961 |  |  |
| Age at Index (mean, SD) | 29.6 ± 6.02 |  | 28.5 ± 6.29 |  | 0.2018 | 29.6 ± 6.02 |  | 29.6 ± 6.06 |  | 0.0010 |
| Not Hispanic or Latino | 673 | 70.031% | 2289018 | 56.566% | 0.2821 | 673 | 70.031% | 674 | 70.135% | 0.0023 |
| White | 626 | 65.14% | 2169123 | 53.603% | 0.2365 | 626 | 65.14% | 631 | 65.661% | 0.0109 |
| Black or African American | 146 | 15.193% | 684965 | 16.927% | 0.0473 | 146 | 15.193% | 143 | 14.88% | 0.0087 |
| ***Comorbidities (n,%)*** | | | | | | | | | | |
| Overweight and obesity (E66) | 95 | 9.886% | 140537 | 3.473% | 0.2589 | 95 | 9.886% | 95 | 9.886% | 0 |
| Nicotine dependence (F17) | 58 | 6.035% | 94108 | 2.326% | 0.1861 | 58 | 6.035% | 60 | 6.243% | 0.0087 |
| Hypertensive diseases (I10-I1A) | 62 | 6.452% | 60786 | 1.502% | 0.2553 | 62 | 6.452% | 62 | 6.452% | 0 |
| Diabetes mellitus (E08-E13) | 31 | 3.226% | 36594 | 0.904% | 0.1637 | 31 | 3.226% | 30 | 3.122% | 0.0059 |
| Chronic kidney disease (N18) | 10 | 1.041% | 4357 | 0.108% | 0.1237 | 10 | 1.041% | 10 | 1.041% | 0 |

**Supplemental Table 6:** Baseline characteristics of analysis of patients with **other polyp of sinus** and patients without T2ID. Baseline characteristics of the cohorts before propensity-score matching and after propensity-score matching. Percentage values refer to the respective groups.

|  | **Before matching** | | | | | **After matching** | | | | |
| --- | --- | --- | --- | --- | --- | --- | --- | --- | --- | --- |
|  | Eosinophilic esophagitis (n) | Eosinophilic esophagitis (%) | No T2ID (n) | No T2ID (%) | Std. Diff. | Eosinophilic esophagitis (n) | Eosinophilic esophagitis (%) | No T2ID (n) | No T2ID (%) | Std. Diff. |
| ***Demographics (n, %)*** | | | | | | | | | | |
| n | 2780 |  | 4311027 |  |  | 2780 |  | 2780 |  |  |
| Age at Index (mean, SD) | 30 ± 6.11 |  | 28.5 ± 6.29 |  | 0.2018 | 30 ± 6.11 |  | 30 ± 6.09 |  | 0.0010 |
| Not Hispanic or Latino | 2229 | 80.18% | 2289018 | 56.566% | 0.5250 | 2229 | 80.18% | 2228 | 80.144% | 0.0009 |
| White | 2363 | 85% | 2169123 | 53.603% | 0.7239 | 2363 | 85% | 2364 | 85.036% | 0.0010 |
| Black or African American | 160 | 5.755% | 684965 | 16.927% | 0.3579 | 160 | 5.755% | 158 | 5.683% | 0.0031 |
| ***Comorbidities (n,%)*** | | | | | | | | | | |
| Overweight and obesity (E66) | 281 | 10.108% | 140537 | 3.473% | 0.2661 | 281 | 10.108% | 282 | 10.144% | 0.0012 |
| Nicotine dependence (F17) | 109 | 3.921% | 94108 | 2.326% | 0.0918 | 109 | 3.921% | 109 | 3.921% | 0 |
| Hypertensive diseases (I10-I1A) | 104 | 3.741% | 60786 | 1.502% | 0.1405 | 104 | 3.741% | 104 | 3.741% | 0 |
| Diabetes mellitus (E08-E13) | 48 | 1.727% | 36594 | 0.904% | 0.0722 | 48 | 1.727% | 47 | 1.691% | 0.0023 |
| Chronic kidney disease (N18) | 10 | 0.36% | 4357 | 0.108% | 0.0522 | 10 | 0.36% | 10 | 0.36% | 0 |

**Supplemental Table 7:** Baseline characteristics of analysis of patients with **eosinophilic esophagitits** and patients without T2ID. Baseline characteristics of the cohorts before propensity-score matching and after propensity-score matching. Percentage values refer to the respective groups.
